# Ideological roadblocks to humanizing dentistry, an evaluative case study of a continuing education course on social determinants of health

**DOI:** 10.1186/s12939-015-0170-2

**Published:** 2015-04-30

**Authors:** Martine C Lévesque, Alissa Levine, Christophe Bedos

**Affiliations:** École de santé publique de l’Université de Montréal, Faculté de médecine de l’Université de Montréal, Montréal, Canada; Division of Oral Health and Society, Faculty of Dentistry, McGill University, Montréal, Canada; Institut de recherche en santé publique de l’Université de Montréal (IRSPUM), Montréal, Canada

## Abstract

**Background:**

Front line providers of care are frequently lacking in knowledge on and sensitivity to social and structural determinants of underprivileged patients’ health**.** Developing and evaluating approaches to raising health professional awareness and capacity to respond to social determinants is a crucial step in addressing this issue. McGill University, in partnership with Université de Montréal, Québec dental regulatory authorities, and the Québec anti-poverty coalition, co-developed a continuing education (CE) intervention that aims to transfer knowledge and improve the practices of oral health professionals with people living on welfare. Through the use of original educational tools integrating patient narratives and a short film, the onsite course aims to elicit affective learning and critical reflection on practices, as well as provide staff coaching.

**Methods:**

A qualitative case study was conducted, in Montreal Canada, among members of a dental team who participated in this innovative CE course over a period of four months. Data collection consisted in a series of semi-structured individual interviews conducted with 15 members of the dental team throughout the training, digitally recorded group discussions linked to the CE activities, clinic administrative documents and researcher-trainer field notes and journal. In line with adult transformative learning theory, interpretive analysis aimed to reveal learning processes, perceived outcomes and collective perspectives that constrain individual and organizational change.

**Results:**

The findings presented in this article consist in four interactive themes, reflective of clinic culture and context, that act as barriers to humanizing patient care: 1) belief in the “ineluctable” commoditization of dentistry; 2) “equal treatment”, a belief constraining concern for equity and the recognition of discriminatory practices; 3) a predominantly biomedical orientation to care; and 4) stereotypical categorization of publically insured patients into “deserving” vs. “non-deserving” poor. We discuss implications for oral health policy, orientations for dental education, as well as the role dental regulatory authorities should play in addressing discrimination and prejudice.

**Conclusion:**

Humanizing care and developing oral health practitioners’ capacity to respond to social determinants of health, are challenged by significant ideological roadblocks. These require multi-level and multi-sectorial action if gains in social equity in oral health are to be made.

## Introduction

Developing approaches to linking research on social determinants of health to clinical practices is considered a key strategy for tackling health inequities, and represents a growing interest in both public health and primary care [1-3]. Proposed reforms, at the global health level, include healthcare service delivery redesign so as to better respond to community needs and expectations [2]. In high income countries, a few initiatives have also aimed to support general practitioners’ capacity to respond to patients’ social problems by facilitating collaboration with community referral specialists or with public health networks, members of which are trained in building cross-sector and multiagency relationships [4].

Notwithstanding their potential to help integrate social determinants into primary care practitioner agendas, a much more fundamental issue must be addressed alongside such collaboration-focused avenues. Research has shown that primary care physicians and other health professionals are lacking in knowledge about and sensitivity to the social problems underpinning biomedical or psychological complaints with which underprivileged patients present [5]. Specifically, the ways in which difficult living conditions and structural factors affect how individuals experience and manage their health are poorly understood by front line providers of care [5,6]. Worse yet, low-income patients are known to encounter prejudicial attitudes and discriminatory behaviors that pose significant access-to-care barriers [7,8].

Research in the field of private dentistry has explicitly revealed differential and discriminatory practices with patients living on social assistance, in part linked to front-office staff and dental professionals’ negative beliefs and prejudice about poverty and welfare [9-12]. In addition to directly compromising access to care, for example when exclusionary practices are adopted, negative patient stereotyping affects the latter’s subjective satisfaction with the clinical encounter and perceptions of service acceptability [13,14]. The dental education literature is replete with calls to develop dentistry students and practitioners’ social responsibility and responsiveness and has emphasized the curricular limits and gaps in preparing future professionals to meet the needs of underprivileged populations [15-19].

In this article, we describe the results of an evaluative case study involving one large private dental clinic in Montreal, Canada. The clinic took part in an original, on-site, and interactive (i.e. discussion based) continuing education course that aims to raise awareness and transfer knowledge on social determinants of health and on their implications for practice. We address the following research questions: 1) How do course participants perceive themselves to have evolved (e.g., regarding beliefs, views, approaches to care etc.) as a result of their participation in the course? and 2) in relation to practice improvements discussed throughout the training (e.g., patient centered principles), what are the perceived barriers to change and objections raised? Transformative effects of the course are the focus of a second article linked to this case study [20]. In this article, we address research question 2. We present and critically reflect upon participants’ perceptions of obstacles to making practice changes, reflective of clinic culture and context, and that constrain the humanization of care. We discuss the implications of these results, for health professional educators, health regulatory authorities, and policy-makers with an interest in healthcare equity and access-to-care.

### Innovative continuing education on social determinants

In an effort to respond to social-relational barriers to oral healthcare, McGill University’s Division of Oral Health and Society launched, in 2006, the *‘Listening to Others’* knowledge translation partnership with the Université de Montréal Faculty of Dentistry, the Quebec Dental Orders, and the Collectif pour un Québec sans pauvreté (Québec Anti-poverty Coalition). Participating members (N = 10) have since conceptualized and produced an ethnodrama based short film and nine online pedagogical capsules (for details on the participatory development of these tools, please see Lévesque et al. [18,21]. The latter contain screen-captured presentations of statistical data and theory on social determinants of health, filmed accounts on the part of people living on social assistance, practitioners, and researchers as well as practical suggestions as to how oral health professionals might better take into consideration patients’ social context, within a patient centered philosophy [22].

These educational tools have been integrated into an on-site, credited, interactive continuing education (CE) course for all dental team members. Through team discussions and critical reflection on learning material, the course aims to promote “perspective transformation”, according to Mezirow’s theory of adult transformative learning [23,24]. Such an approach to teaching aims to facilitate the surfacing and critical reflection on “meaning perspectives”—frames of reference including ideologies, stereotypes, social and moral norms etc. [25]; helping learners test the validity of the premises underlying these belief systems potentially brings about perspective revision and new actions [24]. Through individual or small group coaching and customized practice strategy development, the training is also designed to respond to emerging needs and objectives identified by learners.

## Methods

### Research design: single qualitative case study

Our single qualitative case study [26,27] consists in the participation of a private dental clinic in our CE course, from April to July 2012. The choice of the clinic was both purposive and opportunistic. The dental clinic owner had previously been involved in a pre-test conducted on one of our educational tools; she had shown interest in providing her entire clinic with some training on poverty. Furthermore, the clinic size and composition are typical of Québec private dental practices, thus providing a pertinent context for gaining potentially transferable knowledge. Finally, the case study clinic accepts and treats patients on social assistance within their private practice, and thus met our only inclusion criterion. Ethical approval for the study was issued by the McGill Faculty of Medicine Institutional Review Board, in February 2012.

Case study research is particularly suited to the study of integrated systems—such as a dental clinic—and for achieving in-depth and contextualized understanding of complex processes—such as those involved in transformative learning [26]. The single*-*case design was also coherent with the exploratory and developmental evaluative meta-framework of our research [28].

The participating members of the Montreal based clinic included an all-female staff of 3 dentists, 5 dental hygienists (d.h.), 3 dental assistants (d.ass.), 3 secretaries, and the clinic coordinator. Numbers of years of work experience ranged between 2 and 22. Consistent with the typical set-up of dental clinics in Québec [29], the staff was organized into three dental teams (one per dentist) overseen by the clinic owner, also a practicing dentist. All clinic staff members were wage-earning employees, with the exception of the dentists, two of whom received percentages of the revenues they, or the dental hygienists they supervised, generated. Though there were two staff departures, two employees (one d.h. and one d.ass.) on maternity leave at the time of the training agreed to meet with the researcher-trainer, in a separate small-group setting, to view and discuss the course material. Thus, a total of fifteen team members, all of whom gave informed consent, participated in both the course activities and the associated research process.

In all, our researcher-trainer (first author) provided 8 hours of credited, formal, educational activities, with a majority of the dental team. These activities included group viewing and critical discussion of the fifteen-minute film (2 hour activity) and six pedagogical capsules (5 hours of activity) focused on various social determinants of health (e.g., housing and food insecurity, welfare and work, access to dental insurance, stigmatization). The group activities took place evenings, after clinic hours, so as not to interfere with patient scheduling. Despite staff frustration with this evening schedule, reported fatigue, and the sense that more time would have been required to delve into the topics, group discussions were very dynamic, with a majority of the staff participating throughout.

Three weeks of participant observation also took place in the reception area of the clinic where critical discussions unfolded on occasion with front office staff. However, formal coaching with professional staff did not take place due to time constraints.

### Data collection and analysis

Data collection and analysis were interwoven with course components and included digitally recorded brief (10–20 minute) initial interviews with all participants. These aimed to uncover their baseline perceptions of challenges and successes in working with patients insured through welfare. Two group discussions, that following the film projection and that surrounding the second evening presentation of educational capsules, were also digitally recorded and transcribed. They document group interactions and responses to critical questions on their reactions to the educational material.

Non-directive brief “mid-term” interviews (5–20 min) conducted with 11 participants, and individual semi-structured final interviews (60 to 120 min) conducted with all 15 participants, were equally digitally recorded and transcribed. These recorded aspects of participants’ learning processes and perceived changes in their views and experiences with patients. The first final interviews guided the subsequent ones as adjustments were made to the interview guide according to emerging patterns and themes in the data, consistent with the iterative nature of qualitative data collection and analysis (Please see Appendix 1 for initial final interview guide) [30]. Finally, researcher field notes and a reflexive journal (105 single-spaced pages), as well as several clinic administrative documents (e.g., policy & procedure) were also collected. See Table 1 for a summary of continuing education and research phases, and associated data sources.

**Table 1:** **Phases of continuing education and research, and associated data sources**

**Phases of continuing education and research**	**Phase 1:** ***Baseline problem identification***	**Phase 2:** ***Group film viewing and discussion*** **(2 hours)**	**Phase 3:** ***Audiovisual capsule viewing and discussions*** **(5 hours)**	**Mid- course evaluative research phase**	**Phase 4:** ***Informal coaching*** **(at reception desk)**	**Final evaluative research phase**
	**(April 5** ^**th**^ **–April 29** ^**th**^ **)**	**(May 2** ^**nd**^ **)**	**(May 31** ^**st**^ **and June 21** ^**st**^ **)**	**(June 6** ^**th**^ **-June 12** ^**th**^ **)**	**(June 27** ^**th**^ **–July 18** ^**th**^ **)**	**(September-November)**
Digitally taped brief staff interviews	X (N = 15)					
Field notes	X	X	X	X	X	
Digitally taped group discussion		X	X			
Clinic administrative documents	X					
Digitally taped in-depth semi-structured interviews				X (N = 11)		X (N = 15)
Trainer/researcher diary	X	X	X	X	X	X

The final interviews, group discussions, and clinic documents were entered into QDA Miner qualitative data management and analysis software [31]. Data analysis next took place in recursive stages of data coding and reduction over the course of several months. This included the identification and grouping of meaningful segments corresponding to broad categories associated with perceived course effects (e.g., new beliefs, actions, or experiences with patients) and with perspectives reflective of clinic context and culture interacting with or constraining these effects. A second phase of in-depth analysis involved independent coding of five interviews by the first two authors to refine, at times combine, and adjust the broad categories into more specific emergent themes and patterns. Applying the resulting exhaustive coding scheme to the whole of the data generated a synthesis [32] of participants’ perceived and contextualized responses to the training. Peer debriefing with our third author and triangulation of results with digitally recorded initial and mid-term interviews with participants helped establish the credibility [33] of our findings.

## Results

In response to the continuing education, many participants describe new understandings and increased sensitivity as to the causes of poverty and welfare, aspects of life on welfare, or the impact of poverty on certain patient health behaviors. We elaborate upon these perceived course effects, alongside reported changes in self-awareness and action on the part of certain participants, in a second publication dedicated to this case study [20]. The course and research process also succeeded in uncovering firmly held perspectives that interact among themselves and structure participants’ beliefs, expectations, and feelings about their work with patients living on welfare. These perspectives emerged at times during group discussions, or within interviews, as objections to certain propositional statements made in course material (e.g., in the video capsules). They thus constitute constraints to desirable changes in behavior and action and are presented under the following headings: 1) “Dentistry is a business”; 2) “Equal treatment”, a source of clinic pride; 3) A predominantly biomedical orientation to care; and 4) “Deserving” vs. “non-deserving”, dichotomized thinking about people living on welfare. Interview and group discussion excerpts have been translated from French to English. Pseudonyms are used throughout the results section.

### “Dentistry is a business”

A most powerful constraint to learning concerns the private market context of dentistry and dental team members’ belief in the necessary commoditization of dental care. All participants refer, at some point, to the business, particularly the profitability imperative of their dental clinic. They perceive the ineluctable, dominant and omnipresent influence profitability concerns have on their professional lives. A majority of participants make reference and comparisons to other commodities or services whereas the clinic owner makes analogies between hers and other liberal professions when discussing revenue generation:

*It’s the same for the cashier in a grocery store, her speed and performance are closely monitored… (Sarah, dentist)*

*It’s like if you go to a garage, you won’t get free service… (Sandra, dental hygienist)*

*Lawyers and consultants [similarly] have hourly revenue targets… (Frances, clinic owner)*

Profitability imperatives overtook and superseded implementation of aspects of the training and are also frequently framed as an obstacle to making practice changes.

Course propositions regarding developing conversation with patients on their social context are considered to be in complete conflict with the workflow of a private dental clinic. Despite the fact that the proposed approach is limited to integrating certain key open-ended questions—during initial or annual clinical exams in non-emergency contexts with patients—, the clinic owner feels she cannot afford the time necessary to implement such a shift in clinical practice:

*It’s a question of performance, but there’s also a question of profitability, a business issue… So even knowing ALL of that [knowledge gained on poverty], knowing we should make some changes to our questionnaire, the reality is that it conflicts with the reality of the business of dentistry. (Frances, clinic owner)*

When probed as to whether an assistant or dental hygienist might possibly have more leeway for pursuing questions on patients’ social context, the clinic owner immediately evokes the costs of having her staff take more time to talk with patients.

*There’s a cost to it. Even if it’s someone else [d.h. or dental assistant] who does it, there’s a cost; that person will not be cost-effective. (Frances, clinic owner)*

Business profitability pressures are experienced as both internal and external. The clinic owner considers financial performance and cost-effectiveness essential to meeting the high costs of dental materials and equipment as well as other major clinic overhead such as staff salaries. She also expresses how outside actors—including dental materials firm representatives, business consultants, and accountants—exert pressure on her to increase clinic profits:

*… representatives [from companies and firms], the accountant, the actuary… Firm [representatives] have told me « Jesus, how come you’re not making more money than that! You can’t NOT be making more money than that! » A business is made to be profitable… At one point, you end up realizing that a business is a business, and God knows I learned that painfully… (Frances, clinic owner)*

The clinic owner considers the transfer of financial performance expectations onto her professional staff an essential aspect of increasing their awareness of, sense of motivation, responsibility, and involvement with clinic revenue generation. Though the entire staff consider the profitability imperative as an unavoidable dimension of their work context, a few are very critical of its impact on providing or improving care for patients on welfare. One dentist deplored the inter-dentist financial performance competition she experiences and its consequent disincentive effect on accepting and treating patients on welfare.

*The thing is, they [the other dentists] talk about it… “I billed for such and such amount this morning”… And I’m there, seeing a lot of patients on welfare, and jeez, don’t I feel like crap! Even if I say to myself, hey Sarah, you’re a good dentist, your patients like you, they come to see you, like it or not, you compare yourself to the others… Even if I say to myself, forget about what she said, don’t be competitive, just do your job, like it or not I do it subconsciously: Ok, let’s go, let’s bill, let’s bill, so I’m not worse than them… (Sarah, dentist)*

The three secretaries also bring up how the business dimension translates directly and most significantly into their patient scheduling tasks, which constitute both their chief work stressor and chief source of pride:

*It’s my job to manage the schedule, to find patients for my dentist or for my dental hygienist. It’s my main task… If it’s not done right, I’m the one who is affected, it’s my stress, I’m the one who worries when there’s a hole in the schedule… Dr. Lambert is counting on me: “Laura, she’s a good secretary, she books my schedule.” It’s a little like sales, Martine. (Josie, secretary)*

Preventing missed appointments and filling schedules represent the pinnacle of good secretarial work. All three secretaries consider their scheduling achievements as essential to the survival of the clinic and to maintaining the employment of the entire dental team. Success in these tasks is explicitly recognized by one of the secretaries as closely linked to her job performance evaluation and consequently to her prospective salary increase. The secretaries associate their responsibilities and work conditions with their reactions to patients’ missed or cancelled appointments. Patients insured through welfare, perceived as more prone to repeatedly missing appointments, represent a particular and direct threat to a job well done and worthy of reward:

*We’re all a little involved in the clinic [business]… that has to do with money, that has to do with responsibilities and what has to be done so that the clinic can grow. So people that are on welfare who miss their appointments, that’s why we’re frustrated, us, the secretaries. (Katy, secretary)*

Negative interpretations of and responses to missed appointments on the part of patients on welfare are described as amplified by the entrepreneurial context of the work environment. Secretaries report that pressures associated with scheduling undermine their patience and willingness to empathize:

*If we only have patients on welfare, and they miss their appointments, we won’t go far. And that is why we pass judgment and we don’t think of their problems, us, because we say to ourselves “Hey, I’ve got a schedule to book!” (Katy, secretary)*

All three secretaries equally deplore how their workflow and constant patient turnover at the reception desk compromise both the time and intimacy necessary for developing rapport with patients. They express desire for better and lengthier interactions with patients.

### “Equal care”, a source of clinic pride

A second perspective curbing staff openness to making changes concerns their steadfast conviction of the clinic’s high ethical standard of equal care for all patients. The topic of equal care surfaced in initial interviews, and throughout the training, at times constituting an objection to the course itself: *“I don’t see what it is I have to learn, because I treat all my patients the same way…” (a few participants)*

Participants take great pride in their perceived dedication to equal patient care that they associate with the following: equal time allowed for clinical interventions, equal quality materials and instruments employed, equal dental hygiene education provided, and equal quality performance of dental treatments:

*Whether someone with a lot of cavities is on welfare or not, I will give the same advice*. (Jennifer, d.h.)

*I’m really proud to say we have the best approach I’ve seen, compared to other clinics… I’ve never heard Dr. Frances say: « Let’s decrease treatment time because they’re on welfare. (Cindy, d.ass.)*

*We give them the same quality care… In the [other] clinic where I worked, there were always instruments that weren’t working… It wasn’t quality dentistry… At the end of the day, you really felt that you were botching your work. (Beth, dentist)*

As evidenced above, affirmations on equal care standards at the clinic are closely linked to favorable comparisons to other dental clinics, whether with respect to technical aspects of care, or to the mere equal acceptance, at the clinic, of patients on welfare:

*I don’t really understand all the changes I might make, what I can do more, because, as I said in the beginning, our clinic accepts patients on welfare… I’ve worked in other clinics where it wasn’t like that.* (Beth, dentist)

The equal care moral imperative becomes problematic at times, however, for example when a patient who is insured through welfare requires more in-depth and time-consuming scaling than what is usually provided to patients. A few participants do recognize that their standard of care may not always meet patients’ needs. However, they appear to resolve this “equality” paradox by taking pride in the fact that—“contrary to many other clinics”—they help patients by referring them to alternative resources, dental hygiene schools or university clinics, where the additional care is less expensive than what they would charge the patient.

Statements on equal treatment with respect to relational aspects of care—including how they are welcomed and generally treated—are also at times in stark contradiction with participants’ discourse on specific aspects of their work. For example, the secretaries insist that they equally commend or reprimand all patients, for maintained or missed appointments respectively, yet they admit to taking precautionary measures with patients insured through welfare. These measures include additional warnings and insistence on the cancellation policy with new patients, in an effort to *“*better manage them*”*:

*We tend to infantilize them, “Write it down now…” Because we are so afraid that they will forget us, or they’re people, it seems, that are not with it necessarily; seems like we play a mother role there: “Jot it down, put it on your fridge, there! Don’t forget me now!” Q: How do you feel about this? A: It’s not fun. (Katy, secretary)*

The tendency to differentially infantilize and “warn” patients living on welfare was also reported by other professional staff.

The secretaries also, on the one hand, state that the application of clinic policy with regards to repeat missed appointments (i.e. ‘three strikes, you’re out’) is equal across patient groups. On the other hand, they admit to being more lenient with insured or affluent patients, in particular when expensive treatments are at stake. This practice is considered natural and consistent with the clinic’s profitability imperative:

*A patient on welfare who cancels twice, the third time it’s over, I won’t see him again… I will be a little more flexible with a patient who pays… for sure I’ll give more of a chance to a patient who has a crown on an implant. I think it’s the same for just about everyone [here]. You know, the clinic has to run and all.* (Diane, secretary)

Participants give other examples of differential treatment towards patients on welfare. These include the exclusion from, or requests for justifications for, evening appointments. Others mention strict limitations on longer appointments, expectations regarding patients’ availability to fill in holes in the daytime schedule, and the general tendency for the dentists, over the years, to weed out welfare patients from their caseloads and refer them to newer dentists on staff. These practices are strongly linked to concerns with clinic revenue generation by the participants who, with two exceptions, do not bring up the notion of discrimination or how it might take place in their clinic.

### A predominantly biomedical orientation to care

A third problematic area concerns participants’ dominant perspective on their professional mission as a dental team. During the training, we posited that caring for people living in poverty might include discovering and taking into account their social context. This notion is consistent with most definitions and models of person centered care [22,34], an approach that is gaining ground in dentistry [35]. Yet in response to strategies designed to gain knowledge of a patient’s social context, several participants raise objections that translate a generalized and predominately biomedical orientation to patient care:

*My business is their mouth and teeth. (Cindy, d.ass.)*

*We’re here to serve people with regards to our profession, and to educate them with regards to our profession; so it’s real important that, in our hour of clinical time with the patient, that we talk about dentistry, and not about their financial situation, their socioeconomic difficulty or whatever. (Frances, clinic owner)*

*I find it’s not my role, to ask questions about things like that [transportation to the clinic, housing situation] you know, I’m not there to socialize with them. (Michelle, d.h.)*

The dentists associate patient-centered conversation with “performing social integration”, “mothering”, or “being a psychologist”, all of which they consider incompatible with the typical task demands of the clinical encounter. Furthermore, a few participants attribute any involvement in patients’ social context to the roles of social assistance agents, community workers, or other actors of social and healthcare services.

In spite of course content addressing food insecurity and its implications, regarding the question of food and diet, participants detail how the focus of their care remains on that which is in direct relation to the patients’ teeth and mouth, mostly within the context of preventive education:

*There are certainly people with whom I could do that [discuss access to food and food resources]. But as it is, it’s always just oriented on the mouth you know. (Michelle, d.h.)*

*Q: Do you discuss food and diet with patients? A: Frequently, we make recommendations [on diet] to parents with children with multiple caries… bottle feeding caries… With them, I’ll provide education. (Sandra, d.h.)*

Considering aspects of patients’ lives—whether accessing food or other resources— that are not in direct relation to the mouth or to any oral health problem, represents a new idea for the vast majority of course participants. Not all of them object to this perspective nor discount the feasibility of conversing more with patients on elements of their social context. A few participants indeed feel that attempting to understand patients’ global situation is pertinent and even interesting, in spite of economic constraints.

### “Deserving” vs “non-deserving”, dichotomized thinking about people living on welfare

A final theme having emerged from our study concerns perspectives on patients themselves. Despite having expanded their views, following the course, as to the causes of poverty and the reasons for which people resort to welfare, a majority of participants maintain dichotomized thinking where patients are categorized into two groups: those deserving of welfare, and those not.

*So now I still classify those people into two distinct groups: I find that yes, there are people who really need it [welfare] and it helps them a lot, but I find there are people, I get the impression, that abuse it… (Sandra, d.h.)*

Many participants reasserted their points of view on social assistance categories time and again during the training, frequently to resist addressing or delving into aspects of poverty. In other instances, they tend to question the applicability of issues or course recommendations (e.g., empathizing with social constraints) to certain patients at their clinic. The following post-film group discussion excerpts illustrate these phenomena and the associated inclination, for several participants, to presume ‘guilt’ in absence of ‘proof’ of ‘innocence’.

Excerpt 1: *Diane, secretary: For me, someone who is on welfare, it depends on what it is the person went through… I wonder, for example that woman [the main character in film], why is it that she is on welfare? For me, she should be working, why is she on welfare, why did she end up there? That’s MY question… After that, IF the answer is satisfactory, well, the rest [addressing her social context] I will be ok with.*

Excerpt 2: *Frances, clinic owner: Everyone who is afraid of the dentist or afraid of dental [treatment] costs, will hope their pain is temporary, and tend to wait before consulting for the problem to be constant… I think it’s a human reflex…*

*Beth, dentist: Yes, it’s human… But with welfare [recipients], there are all sorts of categories. There are those that are comfortable on welfare and come here thinking they are the kings of the palace… and there are others…*

The two “welfare patient” profiles respectively comprise very specific and fixed characteristics linked to particular affective responses. The meritorious patient is associated with single parenthood, visible disabilities, and external causal factors (e.g., bad luck) arousing compassion and concern:

*I had another one who came in this week. She is missing a leg… She arrives with all her [welfare] documentation. Amputated, she has all my sympathy! She worked in an office before, caught a virus, and they cut off her leg… I ask her if she needs help, she says “No, it’s ok, I’ve got it…” She touches me, and I feel for her…* (Josie, secretary)

The deserving welfare recipient prototype also refers, for participants, to someone who typically displays shyness or shame, evidence of their psychological struggle with a situation that is unwanted and with which they are unhappy. Their social situation is considered likely temporary. Finally, “meritorious” patients receiving welfare are punctual, do not smoke, are proactive in general and also take measures towards their oral health (e.g., consult preventively, maintain appointments).

*I see that he [the patient] is very very nice, I see that he’s shy about welfare, to show me his [welfare] slip. He is very very shy… and he’s very punctual. (Diane, sec.)*

Much to the opposite, the non-deserving person living on welfare is prototypically content, more or less comfortable with his or her situation, and likely to be abusing the system. Participants attribute moralistic and individualistic causes (e.g., personal choice, laziness) to the situations of people they believe belong to this category, in relation to which they often express contempt:

*… but you know, some people don’t want to work… You know, for them, it’s easier to do that [collect welfare] and work under the table at the same time. (Carol, d.h.)*

*… she’s [a patient] on welfare, she’s content with it [her life], and she likes getting up late. (Diane, sec.)*

Participants also consider that non-deserving patients have more to be blamed for, as they allegedly smoke more or miss appointments without warning, behaviors causing frustration. They are perceived as “nonchalant”, “negligent” or “irresponsible” yet, paradoxically, as more affirmative or “demanding” with regards to their right to public dental care, something that is looked down upon. Patients are identified as belonging to the non-deserving category on the basis of physical attributes or particular behaviors. Those owning trademark possessions, or proposing to pay for certain non-covered treatments (e.g., teeth whitening), are suspected, by some participants, of fraud or system abuse. The same applies to long-term recipients of public assistance or to patients who decline morning dental appointments.

## Discussion

The learning that unfolded within this dental team, through CE on poverty and social determinants, revealed firmly held collective perspectives reflective of the clinic culture, identity, and global societal context. We address and discuss the implications of each of these interacting perspectives that enhance our understanding of the challenges to improving care practices for underprivileged populations.

### “Deserving” vs. “non-deserving”, judgment vs. humanism

Dental professionals’ tendency to categorize patients receiving welfare into deserving and undeserving, or into “good” and “bad” patients, have been documented in previous studies [12,36] and contribute to the very rationale for the course. They are reflective of dominant ideology and societal discourse towards people living in poverty, in particular with regards to beliefs about causality and personal individual responsibility [37,38]. Causal categorization of patients poses a significant challenge, positioning those holding the dichotomized views as judges and moralizers, which is in complete contradiction with the humanistic orientation of our continuing education on social determinants. Maintained causal categorization also restrains, for many, personal engagement with educational material on poverty; conversely, it limits the effects of learning that, though authentic and even profound for some, may remain irrelevant to the care of patients who are considered underserving of benefiting from it. Moreover, knowledge on poverty acquired by people embracing individualist ideology may serve to reinforce—and may even be purposefully sought out to reinforce—arguments supporting such ideology. We address this phenomenon and discuss ways in which the course, and health professional education in general, might tackle categorization more head on, in our second article on this case study. We also reiterate its deleterious effects, and argue that the disqualification of patients by health professionals, as a social determinant of health and mechanism of exclusion, needs to be given more direct consideration by healthcare regulatory authorities. Professional dental boards could, for example, consider campaigning against prejudicial views on publically insured patients, promoting the profession’s moral responsibility in welcoming and treating all members of society regardless of their social status.

### Business vs. humanism

Tensions between the professional and economic regulatory logics inherent to private dental practice have been documented in several studies, and linked to access-to-care and to relationship issues among dentists and low-income patients [39,40]. This case study helps deepen and nuance our understanding of these tensions, their ideological underpinnings, and how they may reverberate across the clinic hierarchy and constrain efforts to humanize care.

Cost-effectiveness and profitability orientations, evidently, come under the responsibility and are at the discretion of the clinic owner. With regards to her perceptions and experience of these financial dimensions of her work, we are struck by the magnitude of the tension and inner conflict they appear to generate. Despite her heightened sensitivity and awareness of difficulties faced by many low-income patients, business imperatives are considered by the clinic owner to considerably limit what she feels able to implement and accomplish, including the types of care models she can afford (biomedical vs patient centered). Cost-effectiveness aside, she associates the economic constraints to making changes with a form of persistent social pressure, to increase profit, coming from the ‘world of dentistry’ (i.e. consultants, industry). Conflict, tension and a diffuse malaise that could be described as restlessness, are also associated with the burden of balancing social and economic interests in Dharamsi’s [40] study of dentists’ perceptions of social responsibility.

Profitability imperatives are integrated, appropriated, and accepted by a majority of our study participants. They mostly do not question or criticize this aspect of their work and a majority also appear to accept the commoditization of dentistry as a given. Yet, as this case study illustrates, the economic logic and associated pressures translate directly into the rapport between staff and low-income patients. It poses a significant roadblock to sensitizing and attenuating the judgment of the secretaries, for example. Their intransigence is manifest regarding patient behaviors (e.g., missing appointments) that compromise the clinic schedule and create a significant source of stress for the secretaries. Given their impact on revenue generation and threat to the secretarial staff’s job performance, these patient behaviors undermine empathy, and elicit or enhance prejudice against patients on welfare. Though we were aware of front office personnel’s tendency to judge Medicaid patients [10] this study reveals the intricate and strong links between staff attitudes towards patients, and issues of self-preservation on the part of the former. Profit-making pressures equally result, according to one dentist, in an atmosphere of competition among her peers, which thwarts her desire to care for patients on welfare. Again, this understanding contributes a new angle from which to consider the many studies documenting dentists’ reticence or refusal to treat patients on welfare [41,42].

In addition to undermining relationships with patients, profitability imperatives contribute to discriminatory practices that are all the more pernicious as they apparently go unrecognized as unfair by many participants. Indeed, many seem to have uncritically adopted practices that they consider to flow directly, and naturally, from the clinic’s economic logic. In several instances, participants comfortably divulge discriminatory treatment all the while resisting any interpretation of their practice as discriminatory.

The implications of these results are multiple. We’ve previously advocated for governments to ensure dental care reimbursement fees that are reasonable, and to extend basic coverage for patients on welfare to include endodontic care, for example [9]. Short of undertaking the even bolder move towards universal public dental care, a highly necessary and specific improvement to current public policy would involve recognition and support for variable patient dental scaling needs. Dentists or hygienists should be allowed to bill for a longer or a second cleaning when this is justified rather than ending the procedure prematurely, which is the current practice and which, of course, amounts to exclusion from care. Such policy improvements would render patients on welfare both more profitable and more satisfying to treat and support the move towards a health equity concept in the care of low-income populations.

However, governments’ involvement and responsibility, as financier, will inevitably fall short, given the high unlikelihood of public funding to ever catch or keep up with a private insurance dominated market [43]. Also, as we have seen through this case study, though essential, sensitivity training is limited in its effectiveness to address practices that are driven by an economic logic that interacts with psychological (e.g., perceived job performance) and social forces (e.g., staff competition, social pressure) that are so strong as to appear, at times, to obliterate social concern.

Given the complex dynamics that contribute to discriminatory practices, diverse approaches and strategies must be set in motion to incite dental professionals to rectify them. Dental regulatory authorities should step in and integrate the evaluation or screening for discriminatory practices within the professional surveillance process. As it is, professional surveillance focuses on aspects such as record keeping, sanitization, waste management, treatment quality, etc. [44] Moreover, the Québec dental code of ethics, art. 2.01[45], stipulates professionals should strive to ensure access to care and public health, yet these issues also somehow elude the surveillance system. Integrating a component on equitable clinic practice and policy would contribute to correcting this situation. Dental authorities should also take advantage of the self-assessment culture that is currently gaining momentum within the realm of health professional licensure maintenance policy and politics [46] and have dentists self-assess for discriminatory practices, from a perspective of continuous quality improvement, and as a condition for maintaining professional licensure. We acknowledge that such action and professional surveillance measures are by no means the guarantor of discrimination-free healthcare environments; however, they would contribute to raising awareness of the issue, and establish dental regulatory authorities’ position against discrimination.

### Biomedical care, a limited model

The next dimension we address relates to the widespread orientation to care that strictly delineates the object of the team’s attention to the teeth and mouth, a dimension that has been embedded in the clinic’s mission statement. The collectively adopted biomedical approach to care that results is particularly unsuited to patients whose lives, behaviors, and logics are frequently and significantly affected by their living conditions and by an array of social determinants. Lack of choice and capacity—whether due to budget, time or psychosocial constraints—to follow professional recommendations will not be properly understood, empathized with, nor taken into consideration, unless addressed within a broader frame of reference. Patient centered care models represent a pertinent alternative because they advocate for a bio-psycho-social understanding of the patient as well as for discovering the meaning the latter ascribes to his or her oral health care issue, within the context of his or her life [22,34,35]. These are indeed keys to sensitive, trust-building, and relevant healthcare for low-income patients.

Patient-centered care is considered foundational to culturally and socially responsive care and is core to many of the practical recommendations nested within our course material. Though many participants developed their sensitivity and understanding of social determinants, our course might have been more effective at leading to knowledge applications if we had better helped surface and reflect critically on the clinic’s collective and unquestioned biomedical philosophy and its impact on relationships with underprivileged patients. These discussions could also have referred more explicitly to the philosophical tenets, in addition to the actual delivery, of patient centered care. Discussion and critical reflection on such topics should also be integral to undergraduate dental education and dental hygiene curricula within an interdisciplinary and holistic approach.

### Equal care standards vs. access-to-care equity

Participants’ collective moral perspective and beliefs regarding equal care at their clinic also raise a few issues. The first concerns the possibility that the unwavering confidence in their “equal care” environment —though mostly associated with technique and quality of clinical care—may contribute to participant’s difficulty recognizing the differential treatment that patients on welfare are subjected to at other levels (e.g., appointment-giving policy).

A second issue refers to the observation that very few participants express their awareness or concern that equal treatment may in fact fall short as a concept for meeting the needs of underprivileged people. We discussed the concrete example of patients at times needing longer cleanings than others, and how providing “equal care” leads to interrupting treatment before its completion; instead of receiving adequate and complete care within the clinic, patients are told to access outside resources. This is a critical area that implicitly challenges the equality credo since equal treatment for unequal needs actually upholds and even deepens inequity.

In response to this second issue, we advance that health professional education, specifically, must help learners deconstruct the equality concept and critically distinguish it from the concept of equity in professional practice. Furthermore, how equitable care might translate into health provider-patient relations and service provision to underserved populations presents an avenue for linking social determinants to individual-level interventions. A pertinent approach for achieving this, as mentioned previously, is for educators to focus on the foundations of patient-centered care, where provider adjustment to individual patient needs implies recognition of their variability [22]. Given the applicability of patient centeredness to patients of all backgrounds and insurance status, its philosophy indulges the professional attachment to the equality ethic all the while implicitly supporting healthcare equity.

Regarding undergraduate dental curricula, efforts should focus on integrating ethical and public health concerns, both transversally and experientially (across the entire curriculum and within practicum) within a patient centered philosophy, as others [47] have advanced. In addition, interdisciplinary and reflection-based approaches to teaching may provide opportunities for critical discussion on the concepts and issues we have addressed. Finally, students must also be engaged in critical analysis of the ways in which the private business-model of dentistry interacts with professional ethics, potentially undermines public health, and may even perniciously generate a sense of legitimacy for discriminatory practices.

## Conclusion

Ultimately, these case study results provide us with an in-depth understanding of important aspects of a private dental team’s response to an educational process aiming to bring about improvements in knowledge, sensitivity, and in practices with underprivileged patients. Our research sheds light on how the study participants consider, experience and attempt to mediate the tensions between their inclination to humanize care and entrepreneurial forces inherent to their private practice context. Though case study results are arguably not generalizable, the knowledge generated may be considered potentially transferable and pertinent to understanding similar healthcare environments and contexts. Moreover, we note the clinic’s resemblance to other Québec private dental clinics in terms of its size and workforce composition.

Our study is, however, not without limitations. First, the all female staff introduces a particularly significant gender bias, given that female practitioners tend to maintain higher levels of empathy throughout their training and upon graduation. One might consequently hypothesize that female practitioners are somewhat more oriented towards patient centered care. Second, participants’ responses in final interviews—conducted by a researcher who was also their course facilitator—are most likely influenced to some degree by social desirability. The latter may have translated into participants’ minimizing or concealing less edifying aspects of their practices and attitudes towards their social assistance patients. Finally, we emphasize how the clinic owner displays a marked level of moral and social concern, as evidenced by her interest and financial investment in the course and in the research. For all these reasons, it is most likely that the reality of prejudice, discrimination, or exclusion of patients living on welfare is harsher in other private dental clinics. In light of this disturbing assessment, we advocate for vigorous and courageous involvement of dental regulatory authorities and health policy-makers in the treacherous fight for healthcare equity in a private for profit care environment.

## Appendix 1: Semi-structured final interview guide (translated from French)

Preambule:

Thank you for agreeing to participate in this interview. This is an important step in my research. One of my main goals is to understand the learning—if any— that took place throughout the continuing education activities. I am interested, for example, in understanding how you perceive the effects the training had on your views and practices with underprivileged patients. Therefore, understanding your perceptions, your opinions, and how you experienced the training are very important to me. Your ideas may also help improve the teaching approach adopted in this continuing education.

1. How would you describe your experience in participating in the course?
  - ➢ What aspects of the course were most significant to you? Why?
  - ➢ Would you say your participation in the learning activities prompted any particular reflections (during or following the training)? If so, please describe.
  - ➢ Would you say you experienced particular emotions or feelings during the learning activities or in relation to them? If so, please describe.
  - ➢ Do you have any suggestions for improving the course, whether in content or form? If so, please share.
2. Do you feel that participating in the course changed something for you? If so, what?
  - ➢ Have you gained any new knowledge as a result of your participation? If so, please elaborate.
    - O with regards to poverty in general
    - O with regards to welfare (e.g., program rules, benefits etc.)
    - O with regards to approaches to care
  - ➢ Has your thinking about patients changed as a result of participating in the course? If so, in what ways?
    - O specifically regarding patients living in poverty
    - O specifically about patients that are living on welfare
    - O specifically about poor (minimum wage earners) workers
    - O with regards to patients in general
  - ➢ Have your view(s) on relationships with patients changed as a result of participating in the course? If so, in what ways?
    - O specifically regarding patients living in poverty
    - O specifically regarding patients that are living on welfare
    - O specifically about poor (minimum wage earners) workers
    - O with regards to patients in general
  - ➢ Has your perception of your role as a dental care professional changed as a result of participating in the course? If so, in what ways?
  - ➢ Has your perception of yourself changed as a result of participating in the course? If so, in what ways?
  - ➢ Have your ways of treating patients or interacting with patients changed? If so, in what way?
    - O specifically regarding patients living in poverty
    - O specifically regarding patients that are living on welfare
    - O specifically about poor (minimum wage earners) workers
    - O with regards to patients in general
  - ➢ Do you perceive any other changes to have occurred in the clinic?
    - O specifically with regards to the physical environment
    - O with regards to staff interactions
  - ➢ In reference to any of the changes mentioned, would you say there were facilitators to making these changes? Please describe.
  - ➢ Are there changes you would like to make or actions you would like to take as a result of participating in the course, but feel you are unable to? How would you describe the barriers to action or change?
    - O at the level of the patient-professional interaction
    - O at the level of the clinic (e.g., organizational issues)
    - O at the level of society
  - ➢ How do you respond to the patient-centered care suggestion of attempting to understand aspects of patients’ social context (i.e. housing situation, level of food security).
